# Cerebrovascular Dysfunction in Alzheimer’s Disease and Transgenic Rodent Models

**DOI:** 10.33696/neurol.5.087

**Published:** 2024

**Authors:** Xing Fang, Fan Fan, Jane J. Border, Richard J. Roman

**Affiliations:** 1 Department of Pharmacology and Toxicology, University of Mississippi Medical Center, Jackson, MS 39216, USA; 2 Department of Physiology, Augusta University, Augusta, GA 30912, USA

**Keywords:** Alzheimer’s disease, Brain hypoperfusion, Beta-amyloid, Tau, Cerebrovascular dysfunction, Neurovascular coupling, AD animal models

## Abstract

Alzheimer’s Disease (AD) and Alzheimer’s Disease-Related Dementia (ADRD) are the primary causes of dementia that has a devastating effect on the quality of life and is a tremendous economic burden on the healthcare system. The accumulation of extracellular beta-amyloid (Aβ) plaques and intracellular hyperphosphorylated tau-containing neurofibrillary tangles (NFTs) in the brain are the hallmarks of AD. They are also thought to be the underlying cause of inflammation, neurodegeneration, brain atrophy, and cognitive impairments that accompany AD. The discovery of *APP, PS1,* and *PS2* mutations that increase Aβ production in families with early onset familial AD led to the development of numerous transgenic rodent models of AD. These models have provided new insight into the role of Aβ in AD; however, they do not fully replicate AD pathology in patients. Familial AD patients with mutations that elevate the production of Aβ represent only a small fraction of dementia patients. In contrast, those with late-onset sporadic AD constitute the majority of cases. This observation, along with the failure of previous clinical trials targeting Aβ or Tau and the modest success of recent trials using Aβ monoclonal antibodies, has led to a reappraisal of the view that Aβ accumulation is the sole factor in the pathogenesis of AD. More recent studies have established that cerebral vascular dysfunction is one of the earliest changes seen in AD, and 67% of the candidate genes linked to AD are expressed in the cerebral vasculature. Thus, there is an increasing appreciation of the vascular contribution to AD, and the National Institute on Aging (NIA) and the Alzheimer’s Disease Foundation recently prioritized it as a focused research area. This review summarizes the strengths and limitations of the most commonly used transgenic AD animal models and current views about the contribution of Aβ accumulation versus cerebrovascular dysfunction in the pathogenesis of AD.

## Introduction

Dementia is an emerging healthcare crisis. Alzheimer’s disease (AD) is the most common form of dementia. There is no cure; current therapies only temporarily improve symptoms but do not prevent progression. One in eight patients >65 and 50% of subjects >85 years old develop dementia [1,2]. Dementia affects one out of every three seniors and is a leading cause of death. The number of deaths caused by Alzheimer’s disease and related disorders (AD/ADRD) annually is higher than the combined deaths from breast and prostate cancer. Midlife hypertension, aging and diabetes are primary risk factors for AD and ADRD [3–9]. Moreover, hypertension has become increasingly prevalent with aging, as demonstrated by its incidence of 60% at midlife and 75% in patients over 70 years old [10]. AD currently afflicts 6.2 million Americans at a cost of $350 billion/yr. The projected outlays for treating AD will rise to 1.3 trillion dollars per year by 2050 [1, 2,11–13]. Thus, there is an urgent need to identify new predictive biomarkers of AD and to introduce new and more effective mechanism-based therapies.

The hallmarks of AD are the accumulation of extracellular beta-amyloid (Aβ) plaques, intracellular hyperphosphorylated tau-containing neurofibrillary tangles (NFTs) and progressive cognitive dysfunction. Although Aβ plaques and NFTs are considered the primary cause of neurodegeneration, inflammation, brain shrinkage, and cognitive impairment in AD, researchers have also observed changes in the microvasculature. Amyloid plaques are formed by extracellular aggregates Aβ, a 40–42 amino acid peptide derived from the amyloid precursor protein (APP), and the accumulation of lower-order aggregates of Aβ contributes to the death of neurons by interfering with synaptic function in critical areas of the brain. NFTs promote neurodegeneration by interfering with the transport of nutrients and essential molecules within neurons.

Familial or early-onset AD is linked to mutations in three genes (*APP, PS1*, and *PS2*). It results in an increased production and accumulation of Aβ in the brain, which can be detected in cerebrospinal fluid (CSF) and by positron emission tomography (PET) imaging [14]. The accumulation of Aβ acts as a central trigger for a series of pathological changes seen in familial AD, including neuronal injury, promotion of the formation of NFTs, and cell apoptosis. However, late-onset or sporadic AD [15] is characterized by decreased Aβ clearance [16]. *APP, PS1,* and *PS2* mutations are rare in late-onset AD patients. This finding has led to questions about whether increased Aβ accumulation is a cause or consequence of AD or if other factors are at play.

Despite intensive study, the cause of late-onset AD is unknown. It is thought to be due to a multifactorial interplay of genetic and environmental factors, including *APOE4* genotype, sex, age, family history, and modifiable factors such as smoking, physical activity, diet, and cardiovascular diseases. Regular exercise reduces the risk of AD, while smoking and a high-cholesterol diet increase the risk of AD. Diabetes, midlife hypertension, and stroke adversely affect CBF, raising the incidence of AD [14].

The accumulation of Aβ and phosphorylated tau proteins in the brain whether due to increased production in familial AD, or reduced clearance in sporadic AD activates microglia and astrocytes, leading to inflammation, microgliosis, and increases in reactive oxygen species (ROS). Elevated ROS increases damage to white matter which can be detected as hyperintensities using magnetic resonance imaging (MRI). Subsequent loss of neurons and other cell types in the cortex and hippocampus leads to cerebral atrophy and cognitive deficits. Thus, AD has traditionally been considered a neurodegenerative disease secondary to excess accumulation of Aβ in the brain. This view led to the development of numerous transgenic animal models of AD with enhanced production of Aβ that have served as the main focus of research in this field for 25 years.

## Transgenic Rodent Models of AD

Most of what is known about the pathogenesis of AD in patients has been derived from behavioral, imaging, and genetic and biomarker studies in AD patients, along with histological studies of postmortem brains. Transgenic rodent models of AD have and continue to serve as the dominant tools for studying pathogenic mechanisms and testing new therapeutic strategies. However, there is no perfect model that recapitulates the full spectrum of AD pathology. Many do not exhibit tauopathy, neurodegeneration, or cerebral vascular dysfunction associated with AD in patients. The strengths and limitations of the most commonly used transgenic animal models of AD are summarized in Table 1 and discussed below.

### 5xFAD mouse model

5xFAD is a well-recognized mouse model used in approximately 10% of AD animal studies [17]. The Swedish (K670N/M671L), Florida (I716V), and London (V717I) mutations in the human *APP* gene and M146L and L286V mutations in *PS1* were introduced into a C57/BL6 mouse in 2006 [18]. The expression of the transgenes is driven by the mouse *Thy1* promoter, which directs the expression of the mutant APP and PS1 to neurons in the forebrain [17]. This model develops amyloid plaques by 2 months of age [19], cerebral amyloid angiopathy (CAA) at 3 months [20], impaired spatial memory at 4–5 months [18,19], loss of synapses at 6 months [21] and a 40% loss of neurons at one-year-of age [22]. This model displays microgliosis and astrogliosis in the cortex and hippocampus by 12 months of age [17]. One-year-old 5xFAD transgenic mice exhibit impaired neurovascular coupling (NVC) responses [23], but cerebral blood flow (CBF) is not reduced, unlike that seen in AD patients [24]. Blood-brain barrier (BBB) integrity and NVC are better preserved in female 5xFAD mice [25] even though Aβ pathology is greater than in males [18]. Thus, although 5xFAD mice display a wide range of AD-like pathologies, they do not fully replicate the disease in humans since they do not develop NFTs and have limited cerebrovascular dysfunction [18,26].

### Tg2576 mouse model

The Tg2576 model is the most well-characterized and widely used AD mouse model. It was developed in 1996 and carries the *APP* K670/M671delinsNL (Swedish) mutation on a mixed C57BL/6 background driven by a hamster prion protein (*PrP*) promoter [27]. Cognitive impairment has been observed in this model as early as 6 months, and CAA first appears at 9 months of age [28]. Aβ plaques and microgliosis can be detected in 10–16-month-old transgenic animals [29]. Impaired functional hyperemic responses first appear around 3 months of age, which precedes amyloid plaque formation [30]. Eleven-month-old Tg2576 mice have an impaired functional hyperemic response to whisker stimulation [31]. Inhibition of plasminogen activator inhibitor 1 (PAI-1) or KO of NOX2 restored NVC and improved cognitive function [32,33]. CBF autoregulation was also impaired in 3-month-old transgenic mice and was correlated with Aβ accumulation in the brain [34]. Unfortunately, this model lacks NTFs and displays minimal neurodegeneration. In addition, the PrP promoter used in this model is now known to direct ectopic Aβ expression in astrocytes, microglia, and oligodendrocytes [35], as well as in the liver, kidney, and spleen [36].

### APP PS1 mouse model

APP PS1 mouse model expresses human transgenes for *APP* with the Swedish mutation and *PS1* with an exon-9 deletion on the C57BL/6J genetic background. The expression is directed to neurons by a mouse *PrP* promoter [37]. Amyloid plaques and plaque-associated astrogliosis and microgliosis can be detected in the cortex by 4 months of age [38–40]. Neurodegeneration is apparent in the striatum in 12-month-old animals [41]. Spatial learning and memory function are impaired in 6-month-old mice, which worsens with age [42]. Impaired functional hyperemia responses to elevated inspired pCO_2_ can be detected at 4.5 months [43]. This mouse model does not develop NFTs and suffers from the same ectopic expression of Aβ protein in other cell types in the brain and systemic organs, as seen in the Tg2576 AD mouse model.

### APP23 mouse model

APP23 mouse model is commonly used to study AD and CAA scince1997 [44]. The model was created to express the human *APP* K670/M671delinsNL (Swedish) mutant gene on the C57BL6 background under the control of the mouse *Thy1* promoter [44,45]. Amyloid plaques appear at 6 months, and leptomeningeal CAA is seen in 9-month animals [44]. CAA progresses to pial, thalamic, cortical, and hippocampal vessels with aging [45]. The accumulation of Aβ leads to disruption of the BBB and cerebral microbleeds [46]. Deficits in spatial memory have been identified in 3-month-old mice [47,48]. Loss of hippocampal neurons (14–28%), microgliosis [49], and synaptic loss [50] have been reported in 14–18-month-old mice [51]. Impaired functional hyperemia responses to whisker stimulation have been seen very early in 3-month-old animals [52]. However, this mouse model lacks NFTs and displays typical long-term potentiation responses throughout its lifetime [53]. Surprisingly, this model exhibits extensive CAA even though a neuronal-specific *Thy1* promoter was used to drive transgene expression.

### 3xTg mouse model

The 3xTg mouse model was generated in 2003 by co-injecting human *APP* (K670/M671delinsNL), *PS1* (M146V) and *MAPT* (P301L) transgenes into a mixed C57BL/6;129X1/SvJ;129S1/Sv genetic background under the control of mouse *Thy1* promoter [54]. The 3xTg model develops prominent Aβ accumulation [55], tau pathology [56] and exhibits learning and memory dysfunction at 6-months. Microgliosis and astrocyte activation are evident by 7 months of age. [57] Microvascular damage has been reported to precede plaque formation at 3-months [58,59]. Neurovascular uncoupling has been reported in 1-month-old transgenic mice associated with decreased NO production [60]. Vessel density is reduced by 35% in 20-month-old 3xTg AD mice versus age-matched controls [61]. Unfortunately, this model shows no evidence of NFTs or loss of neurons.

### J20 mouse model

Two human *APP* mutations, K670/M671delinsNL (Swedish) and V717F (Indiana), under the control of a human platelet-derived growth factor-β (*PDGF-β*) promoter, were introduced into the C57BL/6J genetic background in 2000 [62]. PDGF-β is not specific to neurons but is also expressed in pericytes and immune cells, and ectopic expression of the transgene has been reported in the heart and lungs [63]. Aβ levels are elevated in hippocampal neurons by 6 weeks, and this model develops amyloid plaques at 5–7 months of age [62]. These mice display loss of synapses [64] and neurons in the CA1 region and memory deficits at 3–4 months of age [65,66]. Microgliosis has been observed in 6-month-old J20 mice [65]. At 4-months of age, J20 mice display impaired vasodilatory NO-dependent vasodilator responses of the middle cerebral artery (MCA) to calcitonin gene-related peptides and acetylcholine [67]. Six-month-old J20 mice exhibited an impaired functional hyperemic response to whisker stimulation [68,69] that was reversed by administration of the superoxide dismutase (SOD) mimetic, Tempol [69]. This model mimics most of the pathology in AD patients, except for formation of NFTs.

### TgF344-AD rat model

Rats afford several advantages over mice as an animal model. They are evolutionally closer to humans than mice by several million years [70]. They have larger brains for better image resolution, and more tissue is available for sampling. They are also more robust and amenable to swimming and other behavioral tests. In 2013, Cohen *et al*. [37] generated a human APP-PS1 transgenic rat model (TgF344-AD) of AD by pronuclear injections of a human *APP* with the Swedish mutation and a human *PS1* construct with the ΔE9 mutation under the control of a mouse PrP promoter into Fischer 344 rat embryos. Amyloid plaques, tau pathology, neurodegeneration, and cognitive dysfunction were subsequently followed until 26 months of age [71]. TgF344-AD rats express 2.6 times more APP and 6.2 times more PS1 in the central nervous system (CNS) than control rats. Amyloid plaques appear at 6- months of age. The rats develop tau pathology with NFTs at 16 and 26 months. Microgliosis and astrogliosis become prominent at 6 months of age. There is a 40% loss of neurons in the cortex and hippocampus at 16 months of age. The TgF344-AD rats displayed impaired hippocampal-dependent learning and memory dysfunction at 15 months. They were hyperactive in open field tests, indicating disinhibition associated with hippocampal or cortical injury. More recent studies have indicated that high levels of Aβ oligomers in the brain and spatial learning and memory impairments can be detected as early as 4-months in TgF344-AD rats [72–74].

### Summary

Current rodent models of AD do not fully recapitulate AD pathology in man. Among them, we suggest that Tg2576 transgenic mice and TgF344-AD transgenic rats might serve as the preferred models to study the vascular contribution to AD since they both display cerebrovascular and cognitive dysfunction at an early age, and develop amyloid plaques, synaptic dysfunction, microgliosis, neurodegeneration and cognitive impairments in an age-dependent manner. Although initially developed as familial AD models, the fact that cerebral vascular dysfunction and impaired functional hyperemic responses in these models parallel the expression of Aβ in the cerebral vasculature indicates that they are mixed models, reflective of the majority of AD cases in patients over 65 years of age.

More recently, there has been increasing awareness that vascular risk factors, such as hypertension, diabetes, and atherosclerosis, increase the risk of AD [16,75–77]. These factors damage the cerebral microcirculation leading to reduced CBF and hypoxia, BBB leakage, inflammation, neurodegeneration and the development of vascular cognitive impairment and dementia (VCID) often prior to the detection of Aβ plaques in AD or the relative absence of Aβ deposition in patients and animal models with ADRD.

Several experimental models of ADRD have recently been described. These include carotid artery occlusion models of chronic cerebral ischemia [78,79], chronic hypertension models [7,8,75,80–82], animals fed a high salt diet that activates perivascular macrophages and cerebral ischemia [83,84], pharmacologic blockade of NVC [85], preeclampsia [86–88], models associated with loss of capillary pericytes [89–91], the T2DN diabetic rat model [92–94], a *TgNotch3* mouse model of small vessel disease [95], and Add3 and CYP4A KO models with loss of myogenic tone and autoregulation of CBF [96–100]. These models are associated with some aspects of cerebral vascular dysfunction, including impaired myogenic tone, CBF autoregulation and functional hyperemic responses, reduced capillary perfusion and loss of cognitive function. Interestingly, the loss of cognitive function occurs in the absence of Aβ accumulation and with minimal neurodegeneration or white matter changes. This implies that the cognitive dysfunction is likely due to impaired functional hyperemia and transient hypoxia and may be reversible and treated by drugs that can improve cerebral perfusion.

## Mechanisms Underlying AD Pathology

AD is characterized by the accumulation of extracellular Aβ plaques and intracellular phosphorylated-tau protein, which lead to progressive neuronal loss and cognitive decline [101]. Postmortem, histopathological identification of amyloid pathology remains the gold standard for diagnosing AD. Measurement of the tau/Aβ42 ratio in CSF is increasingly being used as a diagnostic tool and an accepted biomarker for monitoring AD progression in patients. Many mechanisms have been proposed to cause dementia in AD, with the Aβ cascade hypotheses being the most widely accepted. However, mutations that alter the production of Aβ occur in only a small minority of AD patients or those that develop other forms of dementia. Only 24% of patients with dementia have a pure AD pathology (Aβ plaques and NFTs), whereas 80% of clinically diagnosed AD patients exhibit cortical infarcts, microbleeds, and amyloid angiopathy [76,77,102]. Midlife hypertension is the strongest predictor of late-life dementia, and cerebral vascular dysfunction has been identified as one of the earliest pathological changes seen in the development of AD [103,104]. A recent genome-wide association study indicated that 67% of the candidate genes linked to the risk of AD/ADRD are expressed in the cerebral vasculature [105]. Previous studies have also confirmed that cerebral hypoperfusion, BBB leakage, impaired CBF autoregulation and neurovascular uncoupling occur early in the development of AD both in patients and animal models [12,16,76,106]. These findings indicate that there is a close association between AD and vascular pathology and that mixed dementia is the most common form of age-related dementia in elderly patients [16]. However, it remains to be determined whether cerebral vascular dysfunction causes Aβ accumulation, neurodegeneration and loss of cognitive function via focal ischemic damage or is a consequence of Aβ pathology that accelerates the progression of the disease. Below, we have summarized current views regarding the relative contributions of Aβ and Tau accumulation versus cerebral vascular dysfunction to the development of AD and ADRD.

### Beta-amyloid (Aβ) cascade

**Figure 1.**
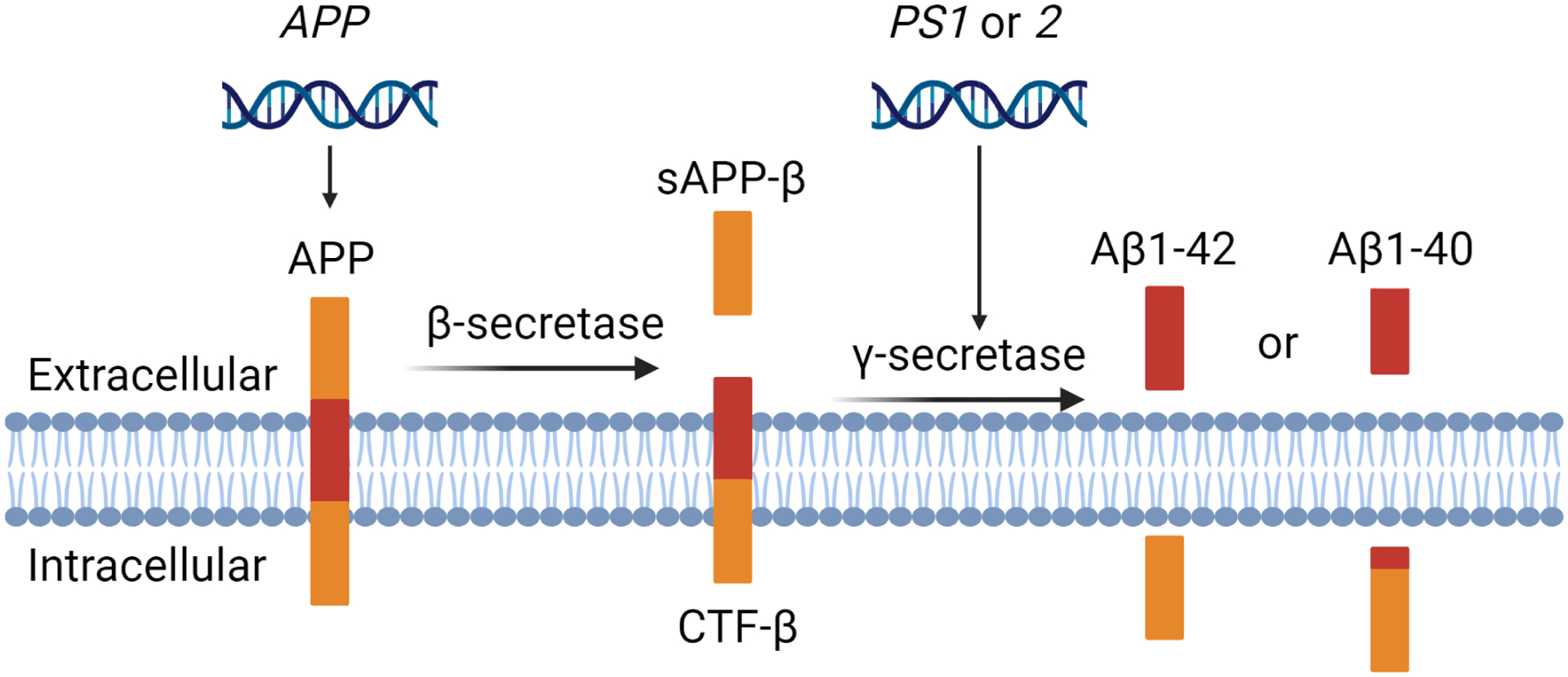
Formation of Aβ peptides via the APP pathway. APP is cleaved by β-secretase into soluble amyloid precursor protein β (sAPP-β) and carboxyl-terminal fragment (CTF-β). The latter is cleaved by γ-secretase into soluble Aβ1–40 and insoluble Aβ1–42 fragments. Presenilin 1 (PS1) or presenilin 2 (PS2) forms the catalytic core of γ-secretase. Mutations in *APP, PS1, and PS2* contribute to the overproduction of Aβ fragments, promoting Aβ deposition in early-onset, familial AD.

Three independent groups first proposed the Aβ cascade hypothesis in 1991 after discovering that Aβ plaques are seen in the brains of all AD patients [107]. The amyloid cascade hypothesis proposed that Aβ accumulation is the primary cause of AD, and produces neurotoxicity and cell death [108]. The Aβ peptides are derived from amyloid precursor protein (APP) (Figure 1), a transmembrane glycoprotein ubiquitously expressed throughout the brain. The most abundantly expressed APP695 isoform is predominately found in neurons [109]. Aβ is produced by proteolytic cleavage of APP by β-secretase-1 (BACE1) at the N-terminus and γ-secretase at the C-terminus [110,111]. It is first cleaved by β-secretase into soluble amyloid precursor protein β (sAPP-β) and carboxyl-terminal fragment (CTF-β). The latter is then cleaved by γ-secretase into soluble and insoluble Aβ fragments. Presenilin 1 (PS1) and presenilin 2 (PS2) serve as the catalytic core of γ-secretase. Mutations in *APP*, *PS1,* or *PS2* are responsible for the overproduction of Aβ fragments in familiar forms of AD [110,112–114]. Insoluble Aβ fragments (Aβ1–42) are deposited in extracellular amyloid plaques, whereas the soluble Aβ fragments (Aβ1–40) aggregate in the perivascular space around arterioles and in the wall of cortical arteries, penetrating arterioles, capillaries, and occasionally, veins [115]. Amyloid plaques exert toxic vascular effects by increasing oxidative stress, channelopathy, inflammation, and cell apoptosis [116]. Aβ accumulation around or in neurons disrupts the electron transport chain in mitochondria [117] and promotes the production of H_2_O_2_ and lipid peroxidation, leading to cellular dysfunction and death [118–123]. Aβ also destabilizes calcium homeostasis in neurons, resulting in excitotoxicity and neuronal loss [124–126]. In addition, Aβ activates microglia and astrocytes, increasing the production and release of proinflammatory IL-1β, IL-6, and TNF-α [127], resulting in neurodegeneration [128]. Aβ also reduces brain-derived neurotrophic factor (BDNF) levels. BDNF is a crucial factor that promotes synaptic plasticity, neuron growth, and survival. Disrupted expression of BDNF causes cell apoptosis, loss of synapses, and neurodegeneration [129]. Increasing evidence also indicates that oxidative stress and inflammation secondary to vascular Aβ accumulation alters cerebrovascular function, contributing to brain hypoperfusion in AD [12,130]. Pericytes enwrapped around the cerebral capillaries play a vital role in regulating capillary perfusion. New evidence has found that Aβ reduces capillary blood flow by increasing oxidative stress and evoking the release of endothelin-1 (ET1) that constricts capillary pericytes [106,131]. Aβ can also promote capillary stalling by increasing the adhesion of neutrophils to the endothelium thereby promoting hypoperfusion in the brain of animal models of AD [132,133].

### Role of Tau

Tau protein is encoded by the gene *MAPT,* which is abundantly expressed in the axons of neurons. Hyperphosphorylation of Tau protein is thought to contribute to the loss of neurons and cognitive dysfunction in AD. Tau protein stabilizes microtubules in neurons to maintain the cytoskeleton that regulates the intracellular transport of nutrients, signaling proteins and transporters [109]. However, Tau protein undergoes abnormal phosphorylation in AD, leading to paired helical filaments leading to the formation of NFTs that disrupt the cytoskeleton. These changes cause neurodegeneration [116] by disrupting mitochondrial integrity [134] and downregulating the expression of BDNF [135]. Additionally, hyperphosphorylated tau protein impairs NVC by decreasing NO production and release from neurons and astrocytes in response to synaptic activity, thereby contributing to impaired NVC.

### AD treatments targeting Aβ pathology

The FDA has approved six drugs to treat AD [14]. Aβ and Tau accumulation in the brain is associated with reductions in cortical cholinergic function [136]. Donepezil, Rivastigmine, and Galantamine are cholinesterase inhibitors that temporarily improve cognitive symptoms in mild to moderate AD by increasing cholinergic transmission [137]. The effectiveness of these drugs varies and is limited in duration. They do not slow the progression of the disease and are associated with significant central and systemic cholinergic side effects [138]. Memantine is an N-methyl-D-aspartate (NMDA) receptor antagonist that prevents overactivity of the glutaminergic system that contributes to neurotoxicity in AD [139]. However, Memantine also has limited efficacy in improving dementia scores using the Mini-Mental State Examination (MMSE) [140].

Two monoclonal antibodies (Aducanumab and Lecanemab) targeting Aβ oligomers were recently approved for use in patients with early-stage AD [141]. Two phase 3 clinical trials investigated the effects of Aducanumab in patients with mild dementia and confirmed amyloid pathology. Both studies found that Aducanumab reduced brain Aβ and Tau levels. Patients treated with high-dose Aducanumab in the EMERGE study exhibited a modest 22% reduction in the decline of cognitive function relative to placebo controls, while the ENGAGE trial failed to detect any reduction in the Aducanumab treated group [141]. Lecanemab was also approved for the treatment of mild AD based on the Clarity AD trial results showing a reduction in amyloid levels over 18 months and a modest reduction in the rate of decline of cognitive function. Similar to Aducanumab, Lacanemab was associated with adverse effects, including headache, confusion, dizziness and more concerning cerebral edema and hemorrhagic amyloid-related imaging abnormalities (ARIA). The mechanism for the development of ARIA is an area of intense investigation. Some investigators suspect that removing amyloid from the wall of vessels may damage cerebral microcirculation, resulting in BBB leakage and microhemorrhages.

## Vascular Hypothesis

### Reduced CBF is an early event in AD

Excess accumulation of Aβ and NFTs is highly correlated with AD progression. However, the failure of clinical trials using β and γ-secretase inhibitors and immune therapies targeting Aβ or Tau [142], as well as the limited success of the recent trials using Aβ monoclonal antibodies to slow the progression of memory loss in patients with AD [141] have led to a reappraisal of the role of vascular dysfunction in late-life dementias [13,16,76,77]. Indeed, 80% of patients with clinically diagnosed AD exhibited cortical infarcts, microbleeds, and CAA [76,77,102].

The most extensive study of the correlation between reductions in CBF and dementia comes from the Rotterdam Study. The results from 1,730 midlife patients with normal cognition showed that a higher baseline CBF was associated with a lower risk of dementia in later-life. Reductions in resting CBF on the order of 10–20% have been found to precede the development of amyloid plaques and the onset of memory deficits in patients who develop AD [13,103,143]. Progression from mild cognitive impairments to frank AD is associated with more global and severe (>40%) reductions in CBF. A summary of some of the studies documenting reductions in CBF in AD patients is presented in Table 2. Prohovnik et al. first reported that cerebral perfusion was significantly lower in AD patients than in healthy controls using a Xenon gas washout technique [144]. Later, several investigators reported reductions in cortical CBF early in the development of AD using single photon emission computed tomography (SPECT) [145,146]. Similar declines in CBF have been seen in various brain regions in AD patients [147], measured by arterial spin labeling magnetic resonance imaging (ASL-MRI) or transcranial Doppler ultrasound [148–158]. Imaging studies have shown that elevated Aβ levels are associated with reduced blood flow in the entorhinal, inferior parietal, and precuneus regions of the brain [148]. The decline in CBF is positively correlated with the loss of cognitive function [104,159,160]. It’s worth noting that vascular dysregulation is an early and persistent symptom of Alzheimer’s disease, which is evident even before the detection of amyloid plaques [103]. Similarly, a decline in CBF has been repeatedly documented prior to the development of cognitive defects or amyloid plaques in multiple transgenic mouse AD models (APP23, J20, APP/PS1, 5xFAD, and Tg2576) that overexpress APP [12,13,16,76,143]. CBF are typically reduced by 10–20% in these AD models [143]. The *APOE4* allele is the most prominent genetic risk factor that increases the incidence of AD in later-life by 3 to 8-fold. Overexpression of hAPOE4 in transgenic knock-in models reduced CBF in the cortex, hippocampus, thalamus and white matter and was associated with loss of pericytes, capillary rarefaction and BBB damage [13,143]. Chronic hypoperfusion has deleterious effects on the brain [13,16,84]. BBB dysfunction and impaired Aβ clearance also contribute to AD pathology associated with vascular dysfunction [12].

### Impaired autoregulation of CBF

CBF autoregulation plays a vital role in maintaining constant blood flow to the brain in response to changes in blood pressure. It involves the complex interplay between the myogenic response (MR) of vascular smooth muscle cells (VSMCs) and pericytes expressing α-SMA, in concert with the release of vasodilatory metabolic mediators from endothelial cells (ECs) and astrocytes [161–164]. Autoregulation of CBF in response to elevations in pressure is partly mediated by constriction of large cerebral arteries and pial arterioles [165–169]. Our group has also recently reported that parenchymal arterioles (PAs) and capillary pericytes also constrict and protect vulnerable capillaries from injury [99,162]. Autoregulation of CBF maintains pressure in PAs and capillaries in the normal range following acute elevations in arterial pressure and the development of hypertension [170], thereby protecting the brain from BBB leakage, edema and inflammation [75,166,168]. However, autoregulation of CBF is often impaired in mouse and rat AD models [16,76,143,171] and hypertensive, elderly, and diabetic patients and animal models [7,13,16,75,94,172]. A recent meta-analysis [173] identified studies showing impaired CBF autoregulation following postural changes in blood pressure in AD patients [174,175]. Increases in the transmission of pressure to the cerebral microcirculation damages capillaries, leading to reduced Aβ clearance, leakage, cerebral edema, inflammation, and neurodegeneration [7,8,75,168,171,176,177]. Elevated capillary pressure is also associated with increased oxidative stress, endothelial dysfunction, and compromised NVC [6,7,12,13,75]. These changes likely explain the strong link between cerebral vascular dysfunction, reduced CBF and cognitive dysfunction with aging [75,178,179]. Taken together, brain hypoperfusion is now considered an early event in the onset and progression of AD, and cerebrovascular dysfunction plays a crucial role in the pathogenesis of AD [13,16,143,180]. Indeed, the NIA-AA Research Framework consortium and the Alzheimer’s Disease Foundation recently recommended that all aspects of aging and cerebrovascular dysfunction should be considered in the diagnosis of late-life dementia and AD [76]. However, the underlying genes and mechanisms for the decline in CBF in AD patients remain unknown and additional studies are needed to determine the mechanisms by which cerebrovascular dysfunction contributes to neurodegeneration and cognitive dysfunction.

### Neurovascular uncoupling in AD

Increases in neural activity increase CBF through NVC. Neurons, astrocytes, capillary ECs, pericytes, and vascular SMCs interact and contribute to NVC responses [130]. In response to elevated neuronal activity, synapses release glutamate, which acts on the NMDA receptors in neurons and astrocytes, causing calcium influx [181]. The increase in intracellular calcium leads to increased production and release of NO [181,182], prostaglandin E_2_ (PGE_2_) and epoxyeicosatrienoic acids (EETs) to relax VSMCs in adjacent arterioles and pericytes on junctional capillaries to increase CBF locally [181]. Inhibitors of the formation of prostaglandins and EETs reduce the functional hyperemic response to whisker stimulation by 20%, while inhibition of the formation of NO decreases the response by 50–60% [183,184].

Capillaries also detect signals from neurons and astrocytes, which is propagated retrogradely along the endothelium to dilate upstream arterioles [12,13,106]. Recent studies have indicated that pericytes that encircle capillaries dilate in response to glutamate, NO, prostaglandin and EETs [13,185]. They constrict in response to hypoxia, norepinephrine and depolarizing concentrations of K^+^ [106,185,186]. Glutamate also stimulates the release of NO and prostaglandins and dilates capillaries near junctional pericytes [185,187,188]. NO synthase inhibitors prevent glutamate-induced capillary dilation [185,188]. Recently, Longden *et al*. [189] reported that elevations in neural activity that increase extracellular K^+^ concentration activate Kir2.1 channels and hyperpolarize capillary endothelial cells [190,191]. The hyperpolarization is transmitted via endothelial cell gap junctions to dilate upstream VSMCs in PAs [189]. Pericytes also constrict and mediate the no-reflow phenomena following ischemia, traumatic brain injury, and cortical spreading depression [13,185,187,192,193]. Loss of pericytes has been associated with capillary rarefaction, cerebral hypoperfusion, and BBB leakage in various animal models and AD patients [13,89,106,186,187,192,193]. Overall, the current view is that about half of the functional hyperemic response is mediated by the dilation of pial arteries and parenchymal arterioles secondary to the release of PGE_2_, EETs and NO from neurons and astrocytes in active areas of the brain. The remaining response is mediated by the activation of Kir2.1 channels in capillary ECs and retrograde transmission of the hyperpolarizing signal to upstream arterioles.

Neurovascular uncoupling and impaired functional hyperemia responses have been reported in AD patients (Table 3). Investigators have found decreases in the CBF responses in the frontal and parietal cortex to increased inspired PCO_2_ and during a verbal fluency task in AD patients compared to healthy elderly controls [194]. Similarly, AD patients have significantly smaller CBF responses in response to visual stimulation or a 5% CO_2_ challenge using PET scans [195], transcranial Doppler ultrasound [174,196] or blood-oxygen-level-dependent functional magnetic resonance imaging (BOLD-fMRI) [197–199] or during a memory encoding task using fMRI [200,201].

Impaired NVC and functional hyperemic responses have been reported in nearly all of the transgenic APP mouse models of AD using laser Doppler flowmetry or multiphoton microscopy [28,31,68,202]. Our lab has also recently reported that TgF-344 AD rats exhibit compromised functional hyperemia in response to whisker stimulation at 4-months of age, two months earlier than the appearance of cognitive impairments or amyloid plaques [203]. The impairment in NVC was associated with reduced expression of Kir2.1 channel protein in cerebral capillaries. These results are consistent with our finding that the vasodilatory response of pre-capillary PAs to local administration of 10 mM KCl to attached capillaries was impaired in this model. Altered CBF hemodynamics and reduced functional hyperemic responses might directly contribute to the loss of cognitive function independent of neurodegeneration early in AD development via cerebral hypoperfusion. Indeed, Tarantini et al. pharmacologically disrupted NVC in normal mice using inhibitors of epoxygenase, cyclooxygenase, and nitric oxide synthase for three weeks and found that impaired NVC was correlated with poor spatial and recognition memory and motor performance [204]. The cause-and-effect relationships between the impaired NVC and cognitive dysfunction were later confirmed in subsequent studies showing that chronic antioxidant therapy restored NVC responses in this model and rescued cognitive impairments in mouse models of AD [85,205,206].

### BBB dysfunction in AD

Breakdown of BBB is also a significant event in AD. BBB biomarkers are elevated in early in the development of AD in patients and animal models, before the appearance of amyloid plaques and tau pathology [13,207]. For example, Van de et al. demonstrated significant BBB leakage in the cortex in patients with early AD [208,209]. Montagne et al. reported an age-dependent decline in the integrity of the BBB in the hippocampus of AD patients [207]. BBB leakage is also greater in individuals with mild cognitive impairment, and there is a clear correlation between cerebral vascular damage with BBB, as indicated by the measurement of pericyte biomarkers in cerebrospinal fluid analysis [210]. MRI and PET studies have revealed the presence of cerebral microbleeds in patients with early AD [211–214]. Post-mortem analyses of the brains of AD patients, especially APOE4 carriers, have indicated higher concentrations of blood-derived proteins such as fibrinogen, prothrombin, plasminogen, immunoglobulin G, and albumin in the hippocampus and cortex [192,215–218]. The accumulation of serum proteins is associated with elevated capillary metalloproteinase-9 (MMP-9) levels and loss of pericyte coverage on capillaries [192,215]. The glucose transporter 1 (GLU1), which plays a crucial role in glucose uptake into the brain, is reduced in the cerebral capillary endothelium of AD patients [219]. Moreover, the function of the P-glycoprotein (Pgp) efflux transporter of drugs and Aβ proteins, has been found to be diminished in AD patients using PET imaging [220]. These findings confirm the contribution of compromised blood-brain transport mechanisms in AD.

Previous studies have employed the APP, PS1, Tau, and APOE4 transgenic rodent AD models to better understand the role of BBB in the pathophysiology of the disease. A recent comprehensive review suggests that the elevation in BBB leakage in the various rodent models of AD is linked to a loss of endothelial cell tight junctions, capillary endothelial cell injury, loss of pericytes and activation of glial cells and perivascular macrophages [221]. BBB dysfunction is associated with a cascade of events involving neurotoxicity, neuroinflammation, and oxidative stress. These alterations, in turn, contribute directly or indirectly to the disturbed Aβ clearance in the neurovascular unit and across the BBB, thus setting up a vicious cycle for further vascular damage and AD pathology.

## Mechanisms Contributing to Cerebrovascular Dysfunction in AD

Neurons, astrocytes, capillary ECs, pericytes, and VSMs all interact to mediate NVC responses [130]. Multiple mechanisms as summarized below, including deficiencies in the formation and bioavailability of NO, diminished Kir2.1 channel expression and activity in ECs and loss of pericytes and capillaries, have all been suggested to reduce CBF and NVC responses in AD patients and various AD models.

### Nitric oxide

A disruption of NO signaling has been long appreciated in AD [222]. Several mechanisms have been proposed to attenuate the NMDA receptor-NOS-NO pathway in AD. In parallel with cerebral hypoperfusion early in the development of AD, BBB breakdown and endothelial dysfunction have been reported [13]. Disruption of the BBB allows for leakage of albumin, fibrinogen, thrombin, and plasminogen and reduced efflux of Aβ in cerebral microcirculation. Accumulation of Aβ and the systemic proteins triggers the activation of microglia and astrocytes to increase the formation of reactive oxygen species (ROS). ROS impairs NVC by phosphorylating NOS to reduce NO production and by scavenging NO to reduce the bioavailability of NO.

Tissue plasminogen activator (tPA) potentiates the expression of the NMDA receptor and glutamate-stimulated release of NO [223]. Levels of tPA were reduced, and those of the tPA inhibitor, plasminogen inhibitor-1(PAI-1), were increased in a mouse model of AD [32]. Knockout of tPA in normal mice impaired functional hyperemic responses to whisker stimulation [224]. Administration of tPA to Tg2576 AD mice reduced CAA, restored the NVC response to whisker stimulation, and improved cognitive function [32]. These results suggest that a fall in tPA levels in AD may attenuate NVC responsiveness by suppressing NMDA-triggered release of NO in response to elevated neuronal activity [32].

Tau accumulation has also been reported to contribute to neurovascular uncoupling by attenuating NO production [202]. Transgenic mice overexpressing a mutant human tau protein displayed neurovascular uncoupling before the formation of tau tangles and cognitive deficits. The alterations in NVC are attributed to a tau-induced reduction in NOS activity, resulting in a lower production of NO.

Another recently identified contributor to NO deficiency in AD is the overproduction of the mammalian target of rapamycin (mTOR). Inhibition of mTOR activity with rapamycin improved memory loss and reversed cerebral hypoperfusion in the J20 mouse model of AD. The mechanism involved a fall in mTOR-induced phosphorylation of eNOS that impaired NO production. Indeed, inhibition of NOS activity with L-NG-Nitroarginine methyl ester (L-NAME) abolished rapamycin-induced increases in CBF in AD mice [225]. Chronic treatment of J20 mice with rapamycin also restored whisker-stimulated functional hyperemic responses to that seen in WT controls [226]. Similar effects were seen in hAPOE4 transgenic mice chronically treated with rapamycin [227].

### Potassium channel dysfunction

Endothelial cell Kir2.1 channel dysfunction is an early event in mouse models of AD, associated with reduced dilatory response of PAs to potassium and functional hyperemic responses *in vivo* [228]. Inhibition of oxidative stress corrected EC-Kir2.1 channel dysfunction [228]. Capillary EC Kir2.1 dysfunction was found to mediate the impaired functional hyperemic response induced by whisker stimulation and the vasodilator response to the administration of 10 mM KCl to cerebral capillaries in the 5xFAD transgenic mouse model of AD [23]. Exogenous administration of PIP2 to AD mice enhanced capillary EC Kir2.1 activity, and restored the functional hyperemia response to whisker stimulation [23]. Systemic administration of PIP2 also rapidly restored functional hyperemic responses to whisker stimulation in a mouse model of vascular dementia [95]. Activation of PLA2 depletes PIP2 in membrane phospholipids in the cerebral microcirculation, following oxidative stress and inflammation [95]. Thus, strategies to reduce inflammation and oxidative stress would be expected to preserve PIP2 levels and Kir2.1 channel function in AD. Unfortunately, chronic PIP2 treatment has yet to be implemented in AD or other models of dementia. Thus, whether PIP2 can rescue functional hyperemic responses and prevent loss of cognitive function in AD remains to be determined.

### Capillary constriction

Pericytes located on the 1^st^–4^th^ branches orders of capillaries arising from penetrating arterioles can constrict and dilate to regulate capillary perfusion [229]. Nortley *et al*. [106] observed that administering Aβ oligomers reduced capillary diameters by ~25% in human brain cortical slices. More than 80% of capillaries exhibited greater than a 5% constriction. Aβ stimulated ROS formation, which prompted the release of endothelin1 (ET1) that constricts capillary pericytes. Capillary density and diameters decreased in the hippocampus of 4–7 month-old APP/PS1 AD mice near Aβ plaques. Injection of ET1 to the hippocampus near Aβ plaques induced profound constriction of nearby capillaries in AD mice [230]. Moreover, injection of Aβ in the hippocampus of WT mice promoted Aβ influx, rather than efflux, from the plasma to the brain through activation of advanced glycation end products (RAGE) receptors on vessels, thus stimulating the release of ET1, capillary constriction and brain hypoperfusion [231]. These results illustrate that the accumulation of Aβ is a cause and consequence of localized reductions in capillary perfusion in AD.

### Capillary stalling

Recent studies have indicated that adherent neutrophils contribute to reductions in capillary perfusion in AD models. Using 2-photon microscopy, Cruz Hernández *et al*. found increased neutrophil adhesion and stalled capillaries in APP/PS1 and 5xFAD mouse models of AD as they increased in age [133]. A decrease in the glycocalyx coating and negative charge of capillary ECs was proposed to be responsible for the increased neutrophil adhesion in this model [91]. Blocking neutrophil adhesion using an anti-Ly6G antibody reduced the number of stalled capillaries (~60%), increased CBF (~20%), and improved spatial and working memory tasks in the APP/PS1 mouse model of AD [232].

Interestingly, while anti-Ly6G ab improved CBF and cognitive function in 15-month-old APP/PS1 mice, it did not increase in cognitive function in older animals [233]. The same research group later reported that the increases in capillary stalling in APP/PS1 mice could be attributed to vascular endothelial growth factor A (VEGF-A) mediated disruptions of endothelial NOS production and loss of BBB integrity in the blocked capillaries [234]. Another group proposed that vascular oxidative stress, which promotes capillary neutrophil adhesion is responsible for the fall in CBF and memory impairments in 10-month-old APP/PS1 mice [235]. They found that administration of a NOX2 inhibitor significantly reduced capillary stalling and increased RBC velocity in these animals, associated with improved memory [235].

### Capillary rarefaction

Several studies have reported increased capillary constriction and capillary rarefaction in the brains of AD patients [236] and in mouse models [237,238]. APP/PS1 AD mice were found to have reduced PDGFRβ positive pericytes and reduced capillary density in the hippocampus and cortex by the age of 6 months [238]. Tg2576 AD mice at 10-months of age displayed reduced capillary density near senile plaques [238]. However, the mechanisms underlying capillary rarefaction in AD are unknown. Constriction of capillaries and capillary stalling could damage ECs and cause the loss of the structural integrity of the blocked capillaries. Chronic blockage of capillary blood flow may lead to neurodegeneration in the surrounding area and loss of functional hyperemic responses in upstream PAs. More recent work indicates that optical ablation of even a single pericyte causes capillary remodeling and reductions in flow that are more severe in elderly mice [239,240].

## Sex Differences in AD and Cerebral Vascular Function

Epidemiological studies have indicated that women have a higher prevalence of AD, with two-thirds of AD cases residing in women [241,242]. While the prevailing view attributes this discrepancy to women’s longer average lifespan (4.5 years more than men) [243], other factors contribute. These include: 1) women born in the 20^th^ century had lower education levels and incomes than men which contributes to an increasing number of dementia cases [243]; 2) men have a higher cardiovascular death rate in middle age, resulting in a lower rate of dementia in those that survive to an advanced age [243]; 3) women lose the neuro- [244] and vascular-protective [245] effects of sex hormones after menopause; 4) women’s brains are more vulnerable to the effects of AD pathology and a weaker interhemispheric functional connectivity compared to men [241,242]. Despite the notable sex differences in the incidence of AD among humans (>3000 studies in 5 years), there has been a shortage of studies to determine the mechanisms involved. Despite the NIH mandate to study both male and female animals, very little is known about sex differences in the onset and severity of AD in animal models. Fewer than 100 studies have been published in the last 5 years. The lack of study in this area may be due to the increased costs of studying sex differences in aging studies and the difficulties of assessing the influence of the menstrual cycle in premenopausal animals.

Previous studies have revealed marked sex differences in the cerebral vasculature. For example, cerebral arterioles from female animals exhibit differences in the thickness of the vascular wall, elevated baseline myogenic tone but impaired myogenic responses to elevations in pressure, and increased responsiveness to endothelial vasodilators before menopause [245–251]. Given the critical contribution of vascular dysfunction in the pathogenesis of AD, additional studies are needed that focus on sex differences in cerebral vascular structure and function in the pathogenesis of AD.

## Emerging Therapies Targeting Cerebral Hemodynamics

Given the close association of cerebral vascular dysfunction in the pathogenesis of AD, several new therapeutic approaches that augment CBF are being evaluated for the treatment of AD, as summarized below.

### Phosphodiesterase-5 inhibitors

Phosphodiesterase-5 (PDE5) inhibitors are approved to treat erectile dysfunction (ED), benign prostatic hyperplasia (BPH) and pulmonary hypertension (PH). They potentiate the effect of NO to increase cGMP in target tissues [252]. A recent study of 7.2 million individuals found a 69% reduction in the risk of developing AD in patients using the PDE5 inhibitor, Sildenafil [253]. Henry and Pellegrino [254] found that ED, BPH, and PAH patients treated with PDE5 inhibitors have a 64.2%, 55.7%, and 54.0% lower risk of dementia. Administration of PDE5 inhibitor profoundly increases the flow velocity in MCA and posterior cerebral artery while not affecting resting CBF in healthy volunteers [255–257]. Chronic PDE5 inhibitor administration has been reported to reduce Aβ levels, tauopathy and inflammation while increasing CBF and reducing cognitive decline in experimental models of AD [258–260]. To summarize, PDE5 inhibitors are associated with a lower risk of dementia in patients and have beneficial effects in experimental mouse models of AD, supporting the potential use of PDE5 inhibitors for the prevention of AD.

### Soluble epoxide hydrolase (sEH) inhibitors

EETs are cytochrome P450 metabolites of arachidonic acid. EETs are vasodilators that reduce inflammation and oxidative stress. In addition, EETs rapidly cross BBB and modulate Aβ accumulation through multiple pathways [261]. Unfortunately, EETs are rapidly converted by sEH to corresponding inactive diols, thus limiting their beneficial effects. sEH expression is upregulated in the brain of AD patients [262]. sEH expression is also elevated in the brains of transgenic mouse and rat AD models [262,263]. Pharmacological inhibition of sEH in 5xFAD mice and TgF344-AD rats reduced Aβ burden, tau pathology, biomarkers of inflammation, ROS, and endoplasmic reticulum stress and improved cognitive dysfunction [262–264]. Genetic KO of sEH in APP/PS1 transgenic mice with astrogliosis retarded the progression of AD by decreasing inflammation and the production of pro-inflammatory factors [265]. Our lab has recently examined whether inhibition of sEH with TPPU could reduce cognitive impairments by improving cerebral hemodynamics in 6-month-old TgF344-AD rats [266]. TPPU effectively rescued impaired learning and memory defects in this model. The impaired myogenic response of the MCA and CBF autoregulation seen in TgF344-AD rats was normalized by chronic TPPU administration. TPPU also reduced the size and number of amyloid plaques in the cortex and hippocampus, though it had no discernible impact on neuron cell numbers [266]. These results support the potential of using sEH inhibition as a promising therapeutic avenue for AD.

### Sodium-glucose cotransporter-2 (SGLT2) inhibitors

Diabetes is a significant risk factor for AD and is associated with poor outcomes in AD patients [267]. In recent clinical studies in North America [268], Europe [269] and Asia [270], the use of SGLT2 inhibitors in Type II DM patients reduced the incidence of AD. Preclinical studies have shown that SGLT2 inhibitors enhance hippocampal-dependent learning, memory, and cognitive functions in a T2DN-AD mouse model [271,272]. The improvement in cognitive function was associated with lower levels of Aβ and hyperphosphorylated Tau and decreased microhemorrhages, microglial activation and neurodegeneration. Similarly, inhibition of SGLT2 with Luseogliflozin in a rat model of type 2 diabetic nephropathy (T2DN) restored the impaired myogenic responses seen in the MCA and PA, normalized CBF autoregulation, rescued NVC, and lowered BBB leakage, neurodegeneration and cognitive dysfunction [94]. Given the beneficial effects seen in mouse models of AD and the T2DN-ADRD rat model, clinical trials are warranted to assess the effectiveness of SGLT2 inhibitors in AD, irrespective of the diabetic status of the patients.

## Conclusions and Future Directions

Cerebrovascular dysfunction and reduced CBF are now recognized as an early event and play a crucial role in the onset and progression of AD. Reduced cerebral blood flow can lead to decreased oxygen and nutrient supply to brain cells, contributing to impaired function and neurodegeneration. However, the underlying genes and mechanisms responsible have not been fully elucidated. Deficiencies in NVC and functional hyperemia responses in AD are associated with decreased NO production and bioavailability and endothelial cell Kir2.1 channel dysfunction. Increased production and reduced clearance in AD lead to increased accumulation of Aβ, which has direct neurotoxic effects and causes capillary constriction, stalling and rarefaction, further reducing capillary perfusion. Despite the failures of numerous clinical trials focused on reducing Aβ and Tau levels or anti-inflammatory interventions, alternative therapeutic approaches addressing cerebral vascular dysfunction have been largely overlooked.

**Figure 2.**
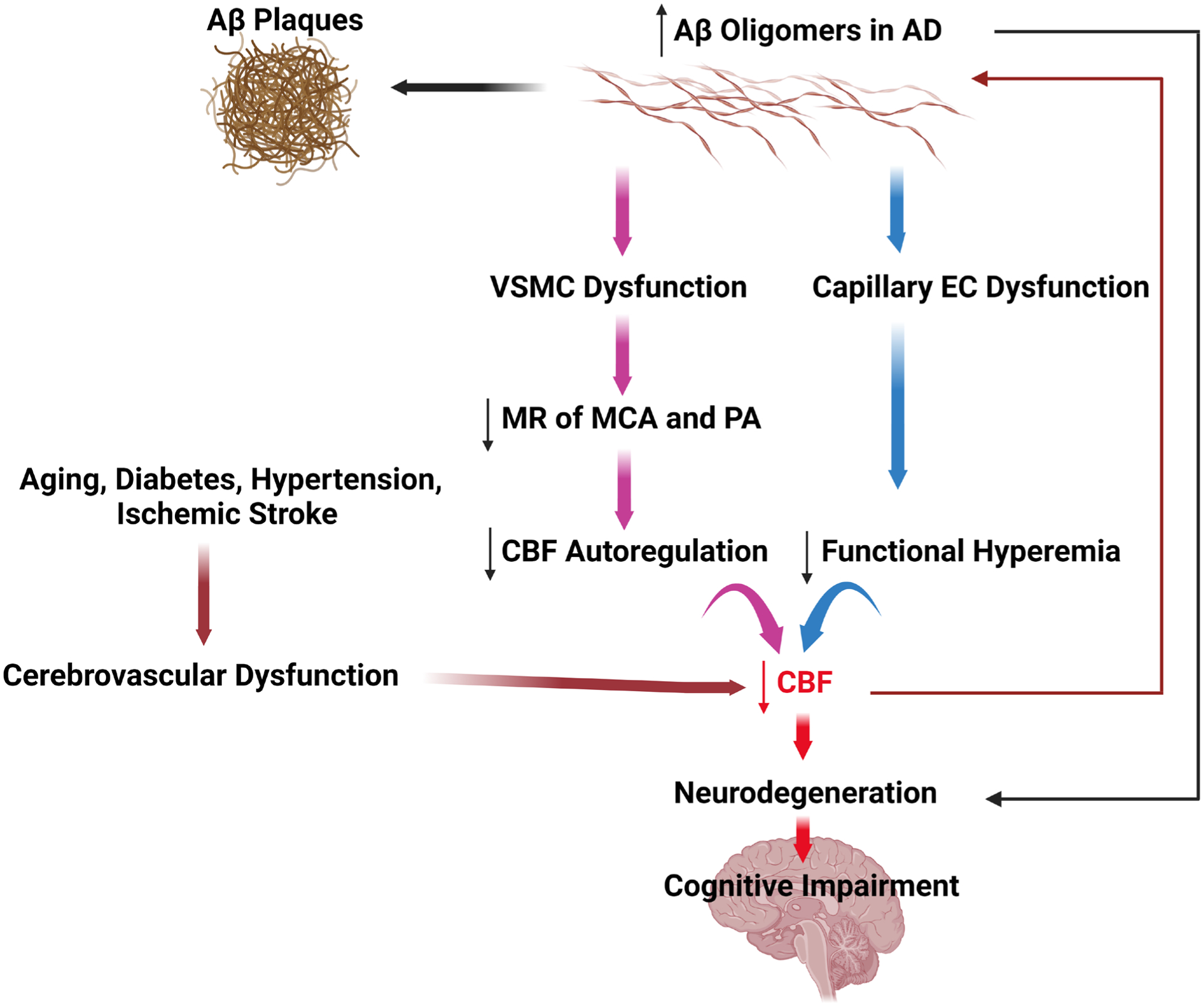
Contribution of cerebral vascular dysfunction in the pathogenesis of AD. Overproduction of Aβ in familial AD leads to vascular smooth muscle cell (VSMC) dysfunction and impaired cerebral blood flow (CBF) autoregulation, and capillary endothelial cell (EC) dysfunction and impaired functional hyperemia, resulting in reductions in CBF, focal ischemia, neurodegeneration and cognitive deficits. Additionally, Aβ has direct toxic effects on neurons and glial cells further contributing to loss of neurons and cognitive impairment. In late-onset sporadic AD, cardiovascular risk factors such as aging, diabetes, hypertension, and ischemic stroke compromise the cerebral microcirculation, leading to reduced CBF, and cerebral hypoxia that impairs neuronal function and cognitive function. In turn, decreased capillary perfusion and hypoxia promote Aβ production and reduces clearance leading to increased Aβ accumulation, creating a vicious cycle for the pathogenesis of AD.

Current evidence supports the view summarized in Figure 2 that excess Aβ production in familial AD adversely affects the structure and function of mural cells and ECs, causing disruptions in CBF regulation, breakdown of BBB, and reductions of brain perfusion. This cascade of events leads to neurodegeneration and the loss of cognitive function due to Aβ toxicity and cerebral hypoperfusion. In Late-onset sporadic AD, risk factors like aging, diabetes, hypertension, diabetes and ischemic stroke gradually decrease cerebral perfusion and Aβ clearance, leading to neurodegeneration and loss of cognitive function via a similar mechanism. Considering the strong evidence that cerebral vascular dysfunction contributes to the pathogenesis of AD, there is now a solid rationale to explore new therapies to prevent damage to the cerebral microcirculation and to restore capillary perfusion in patients with AD and ADRD.

## Figures and Tables

**Table 1.** Strengths and limitations of commonly used rodent models of AD.

Models	Mutations	Promoters	Transgene Expression	Phenotypes	Limitations
***Mouse***					
5xFAD	APP K670/M671delinsNL (Swedish), APP I716V (Florida), APP V717I (London), PSEN1 M146L, PSEN1 L286V	Mouse *Thy1* promoter	CNS neurons	Amyloid plaques, CAA, cognitive impairments, synaptic loss, neuronal loss, microgliosis	Lack of NFTs, limited cerebrovascular dysfunction
Tg2576	APP K670/M671delinsNL (Swedish)	Hamster *PrP* promoter	CNS neurons, astrocyte, microglia, oligodendrocytes; Liver, kidney, spleen	Amyloid plaques, CAA, cognitive impairments, synaptic loss, microgliosis	Lack of NFTs and neuronal loss, Transgene expression beyond CNS
APPPS1	APP K670/M671delinsNL (Swedish), PSEN1 deltaE9	Mouse *PrP* promoter	CNS neurons, astrocyte, microglia, oligodendrocytes; Liver, kidney, spleen	Amyloid plaques, cognitive impairments, microgliosis	Lack of NFTs and neuronal loss, Transgenes expression beyond CNS
APP23	APP K670/M671delinsNL (Swedish)	Mouse *Thy1* promoter	CNS neurons	Amyloid plaques, CAA, cognitive impairments, neuronal loss, microgliosis	Lack of NFTs
3xTg	APP K670/M671delinsNL (Swedish), MAPT P301L, PSEN1 M146V	Mouse *Thy1* promoter	CNS neurons	Amyloid plaques, NFTs, cognitive impairments, microgliosis	Lack of neuronal loss, CAA unknown
J20	APP K670/M671delinsNL (Swedish), APP V717F (Indiana)	Human *PDGF-β* promoter	Predominantly in CNS neurons; Low in heart and lungs	Amyloid plaques, cognitive impairments, synaptic loss, microgliosis, cerebrovascular dysfunction	Lack of NFTs, Transgenes expression beyond CNS
***Rat***					
TgF344	APP K670/M671delinsNL (Swedish), PSEN1 deltaE9	Mouse *PrP* promoter	CNS neurons, astrocyte, microglia, oligodendrocytes; Liver, kidney, spleen	Amyloid plaques, CAA, NFTs, cognitive impairments, synaptic loss, and microgliosis, cerebrovascular dysfunction	Transgene expression beyond CNS

CNS: Central Nervous System; CAA: Cerebral Amyloid Angiopathy; NFTs: Neurofibrillary Tangles.

**Table 2.** **Cerebral blood flow in patients with AD**.

Year	Patients (n)	Controls (n)	Methodologies	Phenotype	Reference
1988	36	12	133Xe inhalation	CBF reduced in AD group	144
1988	16	16	SPECT	CBF in frontal and posterior temporo-parietal cortex in AD group	145
2000	18	11	ASL-MRI	CBF reduced in temporal, parietal, frontal, and posterior cingulate cortex in AD group	151
2002	59	12	SPECT	CBF reduced in temporal, parietal, frontal cortex and hippocampus in AD group	146
2005	20	23	ASL-MRI	CBF reduced in parietal, frontal, and posterior cingulate cortex in AD group	157
2006	10	-	ASL-MRI	CBF reduced in parietal and temporal cortex, precuneus and cingulate gyrus in AD group	155
2006	-	-	Transcranial Doppler	CBF velocity reduced in the posterior cerebral artery in AD group	196
2008	12	20	ASL-MRI	CBF reduced in cingulate, temporal, parahippocampal, and fusiform gyri, thalamus, insula, and hippocampus in AD group	150
2009	19	22	ASL-MRI	Brain perfusion was reduced in the AD group	158
2009	37	38	ASL-MRI	CBF reduced in parietal, frontal, temporal, and orbitofrontal cortex in AD group	149
2013	71	73	ASL-MRI	CBF reduced in precuneus and parietal cortex in AD group	152
2014	17	37	ASL-MRI	CBF lower in cingulate gyrus, precuneus, and occipital region in AD group	154
2014	182	51	ASL-MRI	CBF reduced in throughout the brain in AD patients	148
2016	107	104	ASL-MRI	CBF reduced in advanced AD	153
2017	74	62	Phase-contrast MRI	Whole-brain perfusion was significantly lower in the AD group	159
2017	97	4636	Phase-contrast MRI	CBF associated with dementia risk	160
2021	5861	4548	Meta-Analysis	CBF reduced in AD	147
2021	156	-	ASL-MRI	Reduced CBF is associated with elevated tau in the entorhinal cortex in AD	156

SPECT : Single-Photon Emission Computed Tomography; ASL-MRI: Arterial Spin Labeled Magnetic Resonance Imaging.

**Table 3.** Reduced functional hyperemia in AD patients.

Year	Patients (n)	Controls (n)	Methodologies	Task	Phenotypes	Reference
1997	19	19	NIRS	Verbal fluence task	Reduced response in frontal and parietal cortex in AD	194
1998	10	19	PET	High frequency visual stimulation	Smaller CBF responses in many brain regions in AD	195
2000	12	10	Functional MRI	Memory encoding task	Reduced brain activation in hippocampus and parahippocampal gyrus in AD	201
2003	9	11	Functional MRI	Memory encoding task	Reduced brain activation in medial temporal lobe in AD	200
2006	-	-	Transcranial Doppler	Visual stimulation	Decreased flow velocity in the posterior cerebral artery in AD	196
2011	9	27	BOLD fMRI	CO2 challenge	Reduced CBF response in AD patients	198
2012	17	17	BOLD fMRI	CO2 challenge	Reduced signal changes in forebrain in AD	199
2014	12	24	Transcranial Doppler	CO2 challenge	Reduced flow velocity in cerebral artery in AD	174
2016	35	29	BOLD fMRI	Visual stimulation	Reduced CBF responses in AD patients	197

NIRS: Near-Infrared Spectroscopy; BOLD: Blood Oxygenation Dependent; fMRI: Functional Magnetic Resonance Imaging
